# Antifungal activity of the volatile organic compounds produced by *Ceratocystis fimbriata* strains WSJK-1 and Mby

**DOI:** 10.3389/fmicb.2022.1034939

**Published:** 2022-10-20

**Authors:** Yang Gao, Huan Ren, Shuqi He, Shanquan Duan, Shijun Xing, Xue Li, Qiong Huang

**Affiliations:** State Key Laboratory for Conservation and Utilization of Bio-Resources in Yunnan, College of Plant Protection, Yunnan Agricultural University, Kunming, China

**Keywords:** antifungal activity, biocontrol mechanism, *Ceratocystis fimbriata*, food control, food preservation, postharvest disease

## Abstract

Microorganism-produced volatile organic compounds (VOCs) are considered promising environmental-safety fumigants in food preservation. In this study, the VOCs from fungal *Ceratocystis fimbriata* strains (WSJK-1, Mby) were tested against postharvest fungi *Monilinia laxa*, *Fusarium oxysporum*, *Monilinia fructicola*, *Botrytis cinerea*, *Alternaria solani*, and *Aspergillus flavus in vitro.* The mycelial growth was significantly inhibited, in particular *M. fructicola* and *B. cinerea* (76.95, 76.00%), respectively. VOCs were identified by headspace solid-phase microextraction coupled with Gas Chromatography–Mass Spectrometry (HS-SPME-GC–MS); 40 compounds were identified. The antifungal activity of 21 compounds was tested by the minimum inhibitory concentrations (MIC) value. Benzaldehyde, 2-Phenylethanol, and 1-Octen-3-ol showed strong antifungal activity with the MIC *in vitro* ranging from 0.094 to 0.284 ml L^−1^ depending on the pathogen tested. The optical microscope showed serious morphological damage, including cell deformation, curling, collapse, and deficiency in mycelial or conidia cell structures treated with *C. fimbriata* VOCs and pure compounds. *In vivo* tests, *C. fimbriata* VOCs decreased brown rot severity in peaches, and compounds Benzaldehyde and 2-Phenylethanol could reduce peach brown rot in peaches at 60 μl L^−1^. The VOCs produced by *C. fimbriata* strain have good antifungal effects; low concentration fumigation could control peach brown rot. Its fragrance is fresh, safe, and harmless, and it is possible to replace chemical fumigants. It could be used as a potential biofumigant to control fruit postharvest transportation, storage, and food preservation. To the best of our knowledge, this is the first report on the antifungal activity and biocontrol mechanism of VOCs produced by *C. fimbriata*.

## Highlights

– This is the first report on the antifungal activity of a single compound of volatile organic compounds (VOCs) produced by *Ceratocystis fimbriata*.
– The production of VOCs is an important biocontrol mechanism of *C. fimbriata.*
– The MICs show the highest inhibitory effects of VOCs produced by *C. fimbriata*.
– Volatile organic compounds produced by *C. fimbriata* strains could effectively control the postharvest disease.
– *Ceratocystis fimbriata* can be used as a substitute for traditional chemical fungicides.

## Introduction

Fungal phytopathogens are a major threat to postharvest fruits, causing significant damage to the quality and huge economic losses in fruit storage and transportation (Hernandez et al., 2019; Papoutsis et al., 2019). Although synthetic fungicides remain the key to effective control of pathogenic fungi (Atmosukarto et al., 2005), resistance, and side effects have increased significantly with the long-term, widespread use of various chemical fungicides, which has aroused public concern about environmental pollution and the risks to human health (Xu et al., 2019). Evidence suggests that microbial VOCs are environmentally friendly and could be used in agricultural practices to prevent fungal pathogen development (Kanchiswamy et al., 2015). VOCs produced by microorganisms are considered promising environmental-safety fumigants for controlling postharvest diseases.

Biocontrol agents (BCAs) as a biofumigant is a specific application in biological control since they are not in direct contact with the pathogen and VOCs production is their only action mechanism. VOCs are carbon-containing compounds with low molecular weight and high vapor pressure (Bennett et al., 2012). VOCs produced by antagonistic microorganisms have shown promise for killing a wide range of pathogens. BCAs inhibit plant pathogens through the secretion of active antimicrobial compounds, hydrolysis by cell wall degrading enzymes, and induction of systemic resistance responses in the host (Hancock, 1997; Ongena and Jacques, 2008). VOCs have several advantages, including the ability to diffuse through space, making it easier for users to operate. VOCs can be easily used in postharvest conditions to control diseases infecting vegetables and fruit during storage or long-term transport without the need to discharge them from the store or container. These VOCs can diffuse in the store’s atmosphere, ensuring appropriate protection of agricultural products at the surface without penetrating inside them (Marzouk et al., 2021). Furthermore, biofumigation with VOCs produced by microorganisms avoids direct contact between fruit and antagonist, resulting in no toxic residues on the fruit surface, ensuring greater consumer safety (Toffano et al., 2017).

With the enhancement of environmental protection awareness and consumers’ increasing requirements for quality of food quality, biological control methods have attracted a higher degree of attention (Dimkić et al., 2022; Ni et al., 2022). Biocontrol microorganisms usually include bacteria, fungi, yeast, and actinomycetes (Sellitto et al., 2021). In recent years, it has been found that some fungi can produce abundant VOCs, which show a variety of biological activities, including antimicrobic, plant growth promotion, and nematocidal activity (Duan et al., 2022; Hu et al., 2022). For instance, Isooctanol produced by *Corallococcus* sp. exhibits antifungal activity in *Fusarium oxysporum* (Ye et al., 2020). 3-Methylbutan-1-ol has suppressed *Botrytis cinerea* growth and infection on tomato fruit at 0.442 ml L^−1^ (Chaouachi et al., 2021). Diacetyl could control grey mold caused by *B. cinerea* with only 0.02 ml L^−1^ (Calvo et al., 2020). 2-Nonanone and 2-Heptanone were proved to have strong antifungal properties in *Fusarium oxysporum* (Wu et al., 2019b). In *Bacillus velezensis* G341, three antifungal compounds, Dimethyl sulfoxide, 1-Butanol, and 3-Hydroxy-2-Butanone (Acetoin) were found as potential BCAs for various plant diseases caused by phytopathogenic fungi (Lim et al., 2017). *Candida pseudolambica* W16 inhibited the mycelial growth and conidia germination of *B. cinerea* and caused abnormalities in mycelium morphology and ultrastructure. Fourteen VOCs were identified with the main compounds being 3-Methyl-1-Butanol and 2-Phenylethanol (Zou et al., 2022).

The *C. fimbriata* strain has been studied for its ability to produce large volatiles. It has been considered a new biological source of aroma-producing strains (Christen et al., 1997). It was found that *C. fimbriata* had broad-spectrum antibacterial activity against a wide range of fungi, bacteria, and oomycetes (Li et al., 2015). Previous research has shown that certain *Ceratocystis* spp. could produce a variety of complex VOCs and the antifungal activity of some compounds has been reported. 1-Butanol exhibited strong fungicidal activity against *Alternaria alternata* and *Alternaria clavatus* (Suwannarach et al., 2013). The antagonistic activity of *Bacillus amyloliquefaciens* DA12 was attributable to Heptanones, and the compound could be used as a BCA to reduce the development of fusarium diseases (Lee et al., 2017).

SPME is a modern sample preparation technique that integrates sampling, pre-concentration, and extraction into a single step. The trapped analytes can be directly introduced to analytical instruments like chromatographic systems. HS-SPME has been extensively developed by Pawliszyn’s group. It has been adapted to GC–MS analyses involving volatile and thermally stable compounds in gaseous, liquid and solid matrices. The advantages of this technique include simplicity, shorter extraction times, accurate analysis, and a solvent-free sampling (Jalili et al., 2020). Also, it considerably reduces the possibility of the decomposition and loss of heat-sensitive compounds during the extraction. This technique is used in a wide variety of fields, ranging from food (Mohammadhosseini et al., 2017), environment (Conrady et al., 2021), agriculture (Mohammadhosseini, 2015b), and medicine (Świądro-Piętoń et al., 2022).

This study assessed the antifungal activity of the VOCs produced by two strains of *C. fimbriata* strain WSJK-1 and Mby. Furthermore, we also aimed to identify the chemical nature of the emitted *C. fimbriata* strains VOCs by using HS-SPME followed by GC–MS. We identified the most predominant VOCs metabolites and their antifungal activity *in vitro*. The VOCs of *C. fimbriata* strains were applied to control the postharvest disease of peach brown rot. To the best of our knowledge, this is the first report on the antifungal activity of a single compound of VOCs produced by *C. fimbriata*.

## Materials and methods

### Fungal materials

C*eratocystis* fimbriata strain WSJK-1 (GenBank No. MN990348) and Mby (GenBank No. KY580883) were isolated from diseased lettuce and pomegranate tree in Kunming City and Mengzi County of Yunnan Province, China, and preserved at the State Key Laboratory for Conservation and Utilization of Bio-Resources in Yunnan, College of Plant Protection, Yunnan Agricultural University. The fungal pathogen strains used in this study were grown on potato dextrose agar (PDA) at 25°C for 10 days (Table 1).

**Table 1:** Test fungi.

Species	Host	Disease type
*Botrytis cinerea*	Strawberry	Post-harvest
*Monilinia fructicola*	Peach	Post-harvest
*Monilinia laxa*	Peach	Post-harvest
*Alternaria solani*	Tomato	Post-harvest
*Aspergillus flavus*	Maize	Post-harvest
*Fusarium oxysporum*	Mango	Soil-borne

### Compounds

All compounds used in this study were obtained from Sangon Biotech Company (Shanghai, China) and the compounds were analytical grade.

### Identification of VOCs

The SPME fiber (DVB/CAR/PDMS, Supelco Inc., United States) was desorbed into the headspace VOCs of the double Petri-dish system and equilibrated at room temperature for 30 min to remove residual compounds before extraction. The extraction process was set to 60 min at 40°C. For peak separation and detection, a gas chromatography system (Agilent 7890B, Agilent lnc, USA) equipped with a DB-Wax column (with 30 m length, 250 μm inner diameter, and 0.25 μm thickness; Agilent) and connected to a triple quadrupole mass spectrometer (Agilent 5975C). The chromatographic conditions were as follows: the injection port was heated at 250°C with helium at a flow rate of 1 ml min^−1^. The GC temperature program was set at 40°C for 5 min and then ramped from 40°C to 200°C at 5°C min^−1^ and held for 5 min, 200–240°C at 10°C min^−1^ finally maintained at 250°C for 5 min. The mass spectrometer was operated in an electron impact (EI) ionization at 70 eV with an iron source temperature of 230°C to scan a mass range from 33 to 350 m/z. A mixture of aliphatic hydrocarbons ranging from C2 to C20 was introduced onto the SPME fiber and injected under the same chromatographic program to calculate the Kovats retention index of each compound (Mohammadhosseini, 2015a). GC–MS results were compared with the National Institute of Standards and Technology (NIST-14) spectral database (United States; Lee et al., 2020). The compounds with more than 90% similarity with reference spectra were selected.

### Antifungal activity of VOCs by *Ceratocystis fimbriata* WSJK-1 and Mby *in vitro*

The dual Petri-dishes method assessed the antifungal activity of WSJK-1 and Mby VOCs. For *C. fimbriata*, 7 days-old culture on PDA was placed at the bottom of dishes. For all pathogens, a plate containing PDA on the top of the double Petri-dishes was inoculated with a plug (diameter = 7 mm) of test fungi. The same double Petri-dishes without *C. fimbriata* was used as control. The two base plates were sealed with parafilm and incubated at 25°C for 7 days. Afterward, the fungal diameter was measured and compared with control plates. The results were expressed as the percentage of inhibition (%) compared to the control (Li et al., 2015). Six plates were used per pathogen and *C. fimbriata*, and the experiment was repeated three times.

### Antifungal activity of single VOCs

#### *In vitro* control

According to the analysis results of HS-SPME-GC–MS, the antifungal activity of *C. fimbriata* pure compounds *in vitro* was determined by the double Petri-dish method. An agar plug (7 mm diameter) from actively-growing margins of fungal colonies (7 days-old culture on PDA) was placed at the center of PDA dishes. The plate was used as the top plate of the double Petri-dish system. A round filter paper (20 mm in diameter) was placed on the PDA culture dish used as the base plate of the dual Petri-dish system and impregnated with different volumes of pure compounds. The compound concentration of 120 ml L^−1^ was used to screen antifungal compounds. To find the MICs for all the compounds to pathogens, set concentrations of pure compounds inside the Petri-dishes varied from 0.005 to 1.89 ml L^−1^ headspace. The top plate was then placed onto the base plate and a sterile filter paper without VOCs was used as control. All plates were sealed with parafilm and incubated at 25°C for 7 days. Afterward, the fungal diameter was measured and compared with control plates. The results were expressed as the percentage of inhibition (%) compared to the control. All treatment groups were measured and calculated using the following formula. Six plates were used per pathogen, and the experiment was repeated three times. The pathogen was inhibited by the compound and observed under the optical microscope (Olympus BX5X, DP78 camera, Tokyo, Japan).

Inhibition (%) = (T_0_-T_1_)/T_0_ × 100%

Where T_1_ is the colony diameter of the treatment and T_0_ is the colony diameter of the control.

#### *In vivo* control

Peaches were bought from the Yunnan Agricultural University market. Select healthy fruits of the same size, and then soak them in 5% sodium hypochlorite for 5 min to surface sterilization. Rinse three times with sterile water to remove residual sodium hypochlorite, and blow dry on a clean bench with a UV light on. Use a sterilized punch and scalpel to punch a wound in the center of the peach fruit (Diameter 7 mm, depth 5 mm). They were inoculated with a PDA plug from *M. fructicola* cultures in the wound of peaches. Four VOCs with strong antifungal activity *in vitro* were used for this experiment. The experiment was divided into two groups, place the inoculated fruit in a 5 L plastic box, then put the sterile filter paper absorbing 100, 300, and 500 μl VOCs into the container. The compound’s headspace concentration in the container was 20, 60, and 100 μl L^−1^, and quickly closed the plastic container with a suitable cover. In another group of experiments, the *C. fimbriata* strain cultured on a PDA medium for 7 days was placed in a plastic container and then covered and sealed. Healthy peaches were used as a control. Then, the box was put into the light incubator (light 16 h, dark 8 h, at 25°C) and taken out for 5 days to measure the lesion size and control effect.

### Statistical analysis

Student’s *t*-test and one-way ANOVA followed by a Tukey’s honestly significant difference (HSD) were used to analyze the results using SPSS statistics software (version 22.0). *p* < 0.05 was considered to be significantly different.

## Results and discussion

### *Ceratocystis fimbriata* had a strong antifungal effect on postharvest pathogens

**Figure 1:**
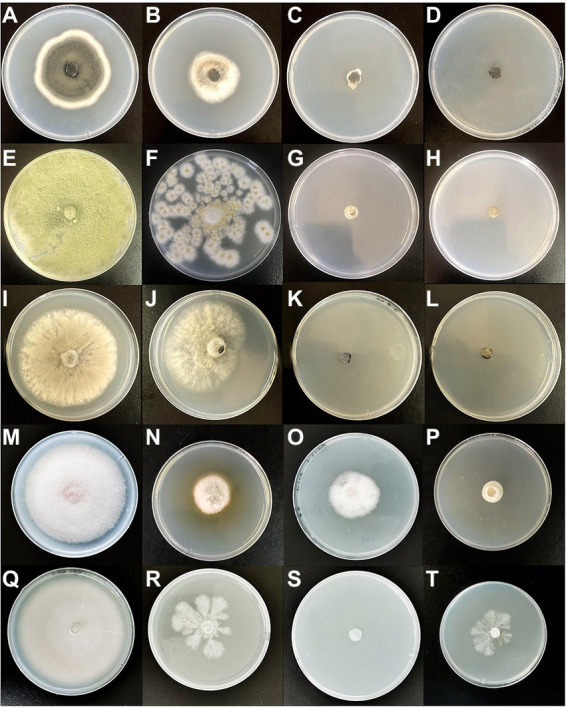
Double Petri-dish assay for antifungal activity of VOCs produced by *Ceratocystis fimbriata*. **(A)** *Alternaria solani* growing on a potato dextrose agar (PDA) medium for 7 days as control. **(B)** *Alternaria solani* treated by *C. fimbriata* growing on a PDA medium. **(C,D)** Treated by 2-Phenylethanol and Benzaldehyde. **(E)** *Aspergillus flavus* growing on a PDA medium for 7 days as control. **(F)** Colony color fades and Area reduction treated by *C. fimbriata*. **(G,H)** *Aspergillus flavus* treated by 2-Phenylethanol and Benzaldehyde. **(I)** *Botrytis cinerea* growing on a PDA medium for 7 days as control. **(J)** *Botrytis cinerea* treated by *C. fimbriata* growing on a PDA medium. **(K,L)** *Botrytis cinerea* treated by 2-Phenylethanol and Benzaldehyde. **(M)** *Fusarium oxysporum* growing on a PDA medium for 7 days as control. **(N)** Extra pigment was produced by *F. oxysporum* treated by *C. fimbriata* growing on a PDA medium. **(O,P)** *Fusarium oxysporum* treated by 2-Phenylethanol and Benzaldehyde. **(Q)** *Monilinia fructicola* growing on a PDA medium for 7 days as control. **(R)** *Monilinia fructicola* treated by *C. fimbriata* was unable normal colony morphology was unable to form, and the colony morphology is the snowflake. **(S)** *Monilinia fructicola* treated by 2-Phenylethanol. **(T)** *Monilinia fructicola* treated by Benzaldehyde normal colony morphology was unable to form, and the colony morphology is the snowflake.

The results were shown in (Table 2; Figure 1) VOCs produced by both strains could inhibit the mycelial growth of all tested pathogens (Table 1). Furthermore, inhibition of mycelial growth from 41.69 to 76.95% was observed by fumigation, indicating that hyphae morphology changed, and the pigmentation became lighter or increased (Figures 1F,N,R).

**Table 2:** *In vitro* antifungal activity of the VOCs produced by WSJK-1 and Mby strains.

No.	Time (days)	Percentage of inhibition (%)					
		*M. laxa*	*F. oxysporum*	*M. fructicola*	*B. cinerea*	*A. solani*	*A. flavus*
WSJK-1	7-day	48.36 ± 0.46d	57.22 ± 1.32c	72.11 ± 0.7a	63.02 ± 1.19b	59.71 ± 0.65bc	55.57 ± 1.14c
Mby	7-day	59.23 ± 1.32c	62.46 ± 0.45bc	76.95 ± 1.03a	76.00 ± 2.2a	70.2 ± 1.01ab	41.69 ± 2.97d

Values represent mean ± SD (*n* = 6). The significant difference (*p* ≤ 0.05) is indicated by the letters using one-way ANOVA followed by Tukey’s test. The treatments are reported only when the differences in means are significant statistically.

Similar to our results, *Trichoderma virens* influenced the morphology of *Rhizoctonia solani* mycelium and inhibited mycelium growth by up to 59.4% (Wonglom et al., 2020). *Aureobasidium pullulans* L1 and L8 strains VOCs significantly inhibited *M. fructigena* mycelium growth (70 and 50%; Di Francesco et al., 2020). The same phenomenon occurs in VOCs produced by bacteria, the B. cinerea mycelium treated with Bacillus amyloliquefaciens BA17 showed swelling and dehydration deformity, protoplast aggregation, mitochondria enlargement and increase, and cell wall defects (Li et al., 2020). The strain Bacillus sonorensis KLBC GS-3 could inhibit mycelia growth of *Penicillium digitatum* and conidial germination *in vitro* (Deng et al., 2020).

### *Ceratocystis fimbriata* contain several antifungal compounds

SPME-GC–MS analysis showed 40 VOCs from 7-day-old *C. fimbriata* WSJK-1 and Mby with ones from the NIST-14 library (Table 3). There were differences in the VOCs between the two strains WSJK-1 23, Mby detected 25 compounds. These compounds mainly fell into several classes: Esters, Aldehydes, Ketones, Hydnocarpates, Acids, Alcohols, Pyrazines, and Alkenes. The major compounds were different in both *C. fimbriata* strains. In WSJK-1, they were Isobutyl acetate (70.61%), Ethyl acetate (11.67%), and Isoamyl acetate (7.58%); in strain Mby, they were, Styrene (24.11%), Ethyl acetate (16.20%), and 1,3-Dimethyl-Benzene (10.34%) of the total VOCs detected.

**Table 3:** VOCs from *C. fimbriata* strains WSJK-1 and Mby produced on 7-day-old PDA culture detected by SPME-GC–MS analysis^*^.

Family	Possible compound	CAS No.	Molecular formula	WSJK-1			Mby		
				Area (%)	CRI^a^	LRI^b^	Area (%)	CRI^a^	LRI^b^
Esters	Ethyl acetate	141–78–6	C_4_H_8_O_2_	11.67	884	887	16.2	885	887
	*n*-Propyl acetate	109–60–4	C_5_H_10_O_2_	0.57	967	972	-	964	972
	Isobutyl acetate	110–19-0	C_6_H_12_O_2_	70.61	1,007	1,011	1.44	1,009	1,011
	Butyl acetate	123–86–4	C_6_H_12_O_2_	0.02	1,060	1,064	-	1,058	1,064
	Isobutyl propionate	540–42–1	C_7_H_14_O_2_	0.13	1,075	1,079	-	1,071	1,079
	Isoamyl acetate	123–92-2	C_7_H_14_O_2_	7.58	1,119	1,123	-	1,116	1,123
	2-Methylbutyl acetate	624–41–9	C_7_H_14_O_2_	3.48	1,122	1,125	-	1,120	1,125
	4-Pentenyl Acetate	1,576–85–8	C_7_H_12_O_2_	0.05	1,200	1,204	-	1,115	1,204
	Benzyl acetate	140–11-4	C_9_H_10_O_2_	0.06	1715	1723	-	1716	1723
				^#^94.17			^#^17.64		
Ketones	Acetone	67–64–1	C_3_H_6_O	0.02	812	817	-	810	817
	2-Butanone	78–93–3	C_4_H_8_O	-	920	925	10.18	922	925
	2-Acetoxyl-3-butanone	4,906–24–5	C_6_H_10_O_3_	2.88	1,370	1,377	1.44	1,372	1,377
	Acetoin	513–86–0	C_4_H_8_O_2_	0.17	1,271	1,277	-	1,269	1,277
	6-methyl-3-Heptanone	624–42–0	C_8_H_16_O	0.01	1,261	1,263	-	1,260	1,263
	2-Heptanone	110–43–0	C_7_H_14_O	-	1,178	1,184	0.86	1,176	1,184
	2-Undecanone	112–12–9	C_11_H_22_O	-	1,588	1,597	0.54	1,585	1,597
	2-Tridecanone	593–08–8	C_13_H_26_O	-	1800	1814	0.57	1798	1814
	2-Tetradecanone	2,345–27–9	C_14_H_28_O	-	1850	1855	0.74	1852	1855
	2-Pentadecanone	2,345–28–0	C_15_H_30_O	-	2011	2021	0.54	2014	2021
				^#^3.08			^#^14.87		
Aldehydes	Benzaldehyde	100–52–7	C_7_H_6_O	0.05	1,501	1,508	5.18	1,498	1,508
	Butanal	123–72–8	C_4_H_8_O	-	845	867	0.7	853	867
	Hexanal	66–25–1	C_6_H_12_O	-	1,086	1,097	8.76	1,088	1,097
				^#^0.05			^#^14.64		
Aliphatics	Ethylene oxide	75–21–8	C_2_H_4_O	0.02	669	680	-	665	680
	Isobutane	75–28–5	C_4_H_10_	0.05	339	354	-	340	354
	Tridecane	629–50–5	C_13_H_28_	-	1,287	1,300	0.21	1,284	1,300
	3-Methyl-5-propylnonane	31,081–18–2	C_13_H_28_	-	1,266	1,268	2.35	1,261	1,268
				^#^0.12			^#^2.56		
Acids	Acetic acid	64–19–7	C_2_H_4_O_2_	0.04	1,445	1,452	-	1,441	1,452
Alcohols	2-Phenylethanol	60–12–8	C_8_H_10_O	0.01	1899	1901	0.76	1894	1901
	3,3-Dimethylbutane-2-ol	464–07–3	C_6_H_14_O	0.05	1,101	1,114	-	1,101	1,114
	Citronellol	106–22–9	C_10_H_20_O	-	1749	1755	3.37	1749	1755
	1-Octen-3-ol	3,391–86–4	C_8_H_16_O	-	1,422	1,430	0.53	1,422	1,430
	2-Tert-Butyl-6-methylphenol	2,219–82–1	C_11_H_16_O	-	1903	1910	2.14	1903	1910
				^#^0.22			^#^6.8		
Pyrazines	Formamide	75–12–7	CH_3_NO	0.07	1786	1791	-	1786	1791
	(S)- (−) Limonene	5,989–54–8	C_10_H_16_	0.05	1,199	1,204	-	1,199	1,204
				^#^0.12					
Alkene	Styrene	100–42–5	C_8_H_8_	0.49	1,253	1,254	24.11	1,253	1,254
	Limonene	138–86–3	C_10_H_16_	0.45	1,176	1,189	-	1,176	1,189
	Ethylbenzene	100–41–4	C_8_H_10_	0.05	1,111	1,123	3.94	1,111	1,123
	1,3-Dimethyl-Benzene	108–38–3	C_8_H_10_	-	1,123	1,143	10.34	1,123	1,143
	*p*-Xylene	106–42–3	C_8_H_10_	-	1,102	1,119	2.76	1,102	1,119
	2-Pentyl-Furan	3,777–69–3	C_9_H_14_O	-	1,201	1,228	1.72	1,201	1,228
				^#^0.99			^#^42.87		

* Compounds present in the control PDA have been subtracted from the data.

a Calculated retention index on DB-WAX column.

b Literature retention index.

# The total percentages of each constituent component in the chemical classification of volatile fractions.

### Antifungal pure VOCs from *Ceratocystis fimbriata* strain

**Figure 2:**
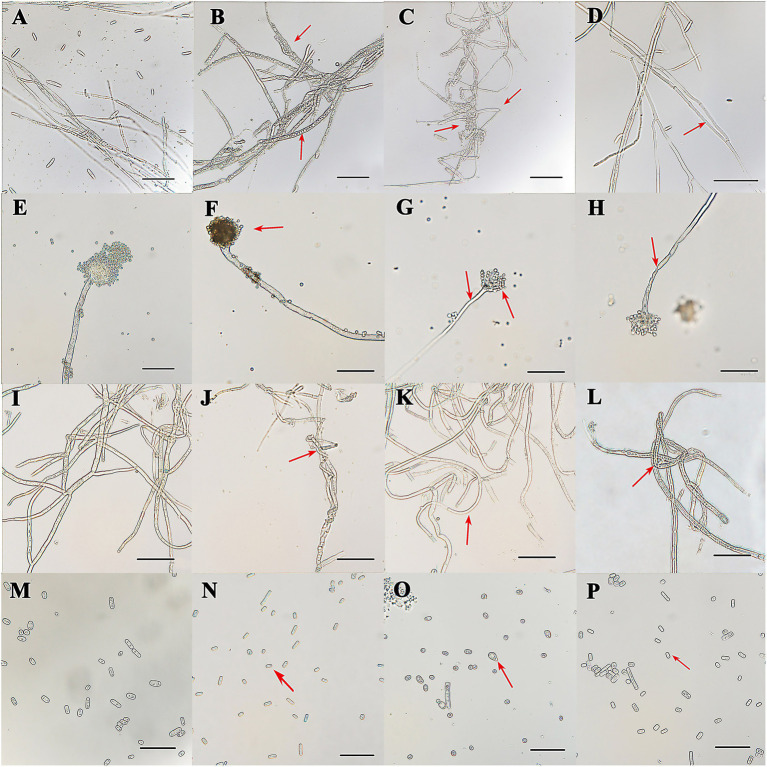
Mycelium and conidia morphology of the pathogen was treated by *C. fimbriata* strain and VOCs from the strain observed under an optical microscope. **(A)** was mycelia and conidia of *F. oxysporum* growing on PDA medium. **(B)** was granulation in the mycelial structures of *F. oxysporum* treated with the strain **(C–D)** was misshapen treated with compounds Benzaldehyde and 2-Phenylethanol in *F. oxysporum.* **(E)** was mycelia and conidia of *A. flavus.* They were growing on PDA medium. **(F)** was misshapen in the mycelial structures of *A. flavus* treated with the strain *C. fimbriata*. **(G,H)** was mycelia collapsed and conidia reduced treated with compounds Benzaldehyde and 2-Phenylethanol in *A. flavus.* **(J,L)** were curling or twisted in *B. cinerea* mycelium treated with *C. fimbriata* strain and 2-Phenylethanol. **(K)** The transverse septae disappeared and mycelium became thinner in *B. cinerea.* **(I)** was untreated control of *B. cinerea* growing on PDA medium. **(N–P)** Misshapen conidium of *M. fructicola* treated with *C. fimbriata* strain, Benzaldehyde and 2-Phenylethanol. **(M)** Normal conidia of *M. fructicola.* All pathogens were fumigated with 0.18 μl L^−1^ compounds or *C. fimbriata* for 7 days. “Scare bar = 50 μm.”

The inhibitions of the single VOCs on six plant pathogens were evaluated (Figures 1, 2; Tables 4, 5). The mycelial diameter was smaller than the control. With the increase of pure VOCs concentration, the colony diameter decreased. The most active compound was Benzaldehyde, which could completely inhibit the growth of *A. solani* mycelium with MICs of 0.094 ml L^−1^. And completely inhibit the mycelium growth of *M. laxa, F. oxysporum, M. fructicola, B. cinerea, A. solani,* and *A. flavus* when the concentration is less than or equal to 0.3 ml L^−1^. 2-Phenylethanol and 1-Octen-3-ol also have high antifungal activity. When the concentration was equal to or lower than 0.189 ml L^−1^fumigation, the mycelium growth of *A. solani* and *B. cinerea* was inhibited. When the fumigation concentration was 0.315 ml L^−1^, all pathogens were inhibited. The inhibitory effects of compounds Limonene (S) - (−)-Limonene, 2-Acetoxyl-3-butanone, 2-Pentyl-Furan, and Hexanal on all pathogens were weaker than Benzaldehyde, 1-Octen-3-ol, and 2-Phenylethanol. The MICs values were equal to or less than 1.89 ml L^−1^ (Table 6). VOCs caused morphological abnormalities in fungal structures according to microscopic observations. Moreover, hyphae’s cell wall and plasmalemma were normal, regular, and homogeneous in control (Figure 2). After being treated with 0.18 μl L^−1^ concentrations of Benzaldehyde and 2-Phenylethanol, the hyphae structures of *F. oxysporum*, *B. cinerea,* and *A. flavus* showed extreme changes, including twisted, curling of fungal mycelium (Figures 2C,J,L), collapsed (Figures 2D,H,G), as well as granulation in mycelial structures (Figure 2B) observed in *F. oxysporum, B. cinerea*, and *A. flavus.* In *B. cinerea*, the transverse septae disappeared (Figure 2K), the formation of conidia in *F. oxysporum* was blocked, and only vegetative mycelium developed (Figures 2B–D). *Monilinia fructicola* was treated with Benzaldehyde and 2-Phenylethanol, and the bubbles appeared in the conidia (Figures 2O,P). In control, the conidia with normal barrel-shaped (Figure 2M).

**Table 4:** Antifungal activity of VOCs by *C. fimbriata*.

Compounds	Inhibitions at Concentration of 2.37 ml L^-1 *^					
	*M. laxa*	*F. oxysporum*	*M. fructicola*	*B. cinerea*	*A. solani*	*A. flavus*
Ethyl Acetate	−	−	−	−	+	−
n-Propyl acetate	−	+	−	+	−	−
Isobutyl acetate	−	−	−	−	−	−
Butyl acetate	+	−	−	−	−	−
Ethyl Acetate	−	−	−	−	−	−
Isobutyl propionate	−	−	−	−	+	−
Isoamyl acetate	−	−	−	−	−	−
Benzyl acetate	−	−	−	+	−	−
2-Butanone	−	+	−	−	+	−
Benzaldehyde	++	++	++	++	++	++
Hexanal	−	−	−	+	+	−
2-Phenylethanol	++	++	++	++	++	++
Acetoin	+	−	+	−	−	+
(S)- (−) Limonene	−	+	+	+	++	+
Styrene	−	−	−	−	−	−
Limonene	+	+	+	+	+	+
1,3-dimethyl-Benzene	−	−	−	−	−	−
Ethylbenzene	−	−	−	−	−	−
1-Octen-3-ol	++	++	++	++	++	−
2-Acetoxyl-3-butanone	−	+	−	−	+	+
2-Pentyl-Furan	−	−	−	+	+	−

“-” represents that the inhibition rate of the compound against pathogens is less than 40% at the concentration of 2.37 ml L^−1^. “+” means that the inhibition rate of the compound against pathogens is less than 70% and more than 40% at the concentration of 2.37 ml L^−1^. “+ +” represents that the inhibition rate of the compound against pathogens is more than 90% at the concentration of 2.37 ml L^−1^.

**Table 5:** *In vitro* MICs of several pure VOCs produced by *C. fimbriata* against tested fungal pathogens.

Compounds	MIC (mL L^−1^of head space) in vitro^*^					
	*M. laxa,*	*F. oxysporum*	*M. fructicola*	*B. cinerea*	*A. solani*	*A. flavus*
Benzaldehyde	0.268	0.300	0.252	0.157	0.094	0.173
2-Phenylethanol	0.284	0.315	0.315	0.189	0.173	0.268
Limonene	-	0.630	-	-	1.26	1.26
(S)- (−)-Limonene	-	1.89	1.89	-	1.89	1.89
1-Octen-3-ol	0.315	0.268	0.157	0.157	0.173	0.300
2-Acetoxyl-3-butanone	0.945	1.26	0.945	0.568	1.89	-
2-Pentyl-Furan	1.26	0.630	1.26	1.26	0.6	-
Hexanal	-	-	-	0.630	1.89	1.89

* (−) Partial inhibition at 1.89 ml L^−1^ (maximum concentration tested).

**Table 6:** Inhibition of *C. fimbriata* and pure VOCs against fungal postharvest pathogens inoculated on Peaches *in vivo*.

Pathogens	Fruits	Compounds	Concentration (μl L^−1^)	Inhibition %
*M. fructicola*	Peach	Benzaldehyde	100	100.00 ± 0.00a
			60	95.89 ± 0.57b
			20	91.38 ± 1.03c
		(S)- (−) limonene	100	34.7 ± 2.97a
			60	11.72 ± 2.64b
			20	7.32 ± 1.47b
		Limonene	100	42.29 ± 1.26a
			60	21.46 ± 4.31b
			20	11.52 ± 2.73b
		2-Phenylethanol	100	100.00 ± 0.00a
			60	86.58 ± 1.32b
			20	75.46 ± 3.68c
		1-Octen-3-ol	100	100.00 ± 0.00a
			60	100.00 ± 0.00a
			20	83.23 ± 1.12a
		WSJK-1	-*	52.31 ± 5.41b
		Mby	-*	63.11 ± 2.93a

* “-” 10 plates of 6-day-old *C. fimbriata* cultures were placed in the boxes.

Values represent mean ± SD (*n* = 3). The significant difference (*p* ≤ 0.05) is indicated by the letters using one-way ANOVA followed by Tukey’s test. The treatments are repeated three times.

*Ceratocystis fimbriata* strains WSJK-1 and Mby contained many antifungal compounds (Table 3), which may be the potential mechanism of the antifungal effect of the strain. The antifungal compounds found included Benzaldehyde, 2-Phenylethanol, 1-Octen-3-ol, Acetic acid, Ethylene oxide, 2-Acetoxyl-3-butanone, 2-Pentyl-Furan, Limonene, and (S) - (−)-Limonene (Chitarra et al., 2010). The mechanism of VOCs as BCAs includes damage to the integrity of cell membranes, leading to the leakage of cell components and oxidative stress (Di Francesco et al., 2016). o-Vanillin was a VOC with an antifungal effect on *A. flavus* that changed the OH functional groups on cell walls and reduced the protein content of mycelial cell walls. In addition, *o*-Vanillin increased the permeability of the cell membranes (Li et al., 2021). *Bacillus* spp. VOCs caused an increase in reactive oxygen species (ROS) in *Sclerotinia sclerotiorum* hyphae cells structural defects in cell walls cytoplasm and multivesicular structures disruption, possibly resulting from oxidative stress (Massawe et al., 2018). Chitin is an important component of the cell wall and plays an irreplaceable role in the cell integrity (Li et al., 2021). Mycelial deformity and diaphragm disappearance after *C. fimbriata* strains fumigation may be related to chitin synthesis. The production of 2-Phenylethanol could affect gene expression and exert its biological control ability (Hua et al., 2014; Loi et al., 2020) And inhibit spore germination and aflatoxin formation in *A. flavus*. and decrease gene expression of clustering aflatoxin biosynthesis genes as evidenced (Di Ciaccio et al., 2020). In the report that 2-Phenylethanol exhibited strong antibacterial activity by breaking the bacterial cell membrane (Monggoot et al., 2016). These data suggest that the mechanism of the antifungal effect of 2-Phenylethanol may be through reducing toxin content and destroying cell membrane integrity. The current study demonstrates that Benzaldehyde has the highest antifungal capacity against fungal pathogens. Effect on fungal of *B. cinerea, M. fructicola, M. laxa, P. italicum*, *P. digitatum*, and *P. expansum* the lowest MIC concentrations, ranging from 0.005 to 0.125 ml L^−1^ depending on the pathogen tested (Calvo et al., 2020). Benzaldehyde inhibits *B.cinerea* binding to G-Protein Coupled Receptors (GPCRs) and transmitting with cyclic adenosine monophosphate (cAMP) -Signal Pathway of the Fungus. One of the identified GPCRs in *B. cinerea*, BcGPR3 bound tightly to Benzaldehyde. Related genes associated with the cAMP signaling pathway were downregulated in *B. cinerea* exposed to Benzaldehyde. The BcGPR3 protein is inactivated by the active compounds and thus fails to transmit signals to the cAMP pathway, inhibiting *B. cinerea* (Lin et al., 2019). Membrane damage resulted in deformed cell structure and the inclusion of organelle material in the cytoplasm, suggesting that cells and organelle membranes could be a potential target for the VOCs emitted by *C. fimbriata*. Because these compounds have been shown in previous findings to permeate and severely damage biological membranes, the presence of 2-Phenylethanol, Benzaldehyde, and 1-Octen-3-ol substances in the VOCs mixture could be the cause of membrane distortion.

However, we have little research on the antifungal mechanism of the mixed VOCs of *C. fimbriata* strain. In the previous reports, *C. fimbriata* strain did not find any compounds related to the antifungal effect, in which esters accounted for the majority. However, the strain still showed an effective antifungal effect (Li et al., 2015). The mechanism of *C. fimbriata* strain mix VOCs needs to be further studied. It has been suggested that VOCs from BCAs usually occur at very low concentrations. Their effects are believed to reflect synergic or additive action rather than the activity of a single component (Di Francesco et al., 2020). Thus, the mechanisms underlying the inhibition of fungal pathogens by VOCs produced by BCAs are seemingly multifactorial and complex.

Using gas-phase compounds with antifungal activity from microorganisms to control plant diseases was one of the applications of biological fumigation. Biological fumigation was first reported in *M. albus* with biocontrol activity against many pathogens (Stobel et al., 2001). Subsequently, VOCs isolated from *M. albus* were successfully commercially applied to inhibit the growth of pathogens in humans, plants and soil, as well as the storage and transportation of fruits (Strobel, 2006; Saxena and Strobel, 2020). VOCs from *Trichoderma* spp. have been formulated commercially in the biocontrol (Siddiquee and Shafiquzzaman, 2014). Moreover, *Trichoderma*’s antifungal activity is associated with a VOC named 6-Pentyl-*α*-Pyrone (Taha et al., 2020).

**Figure 3:**
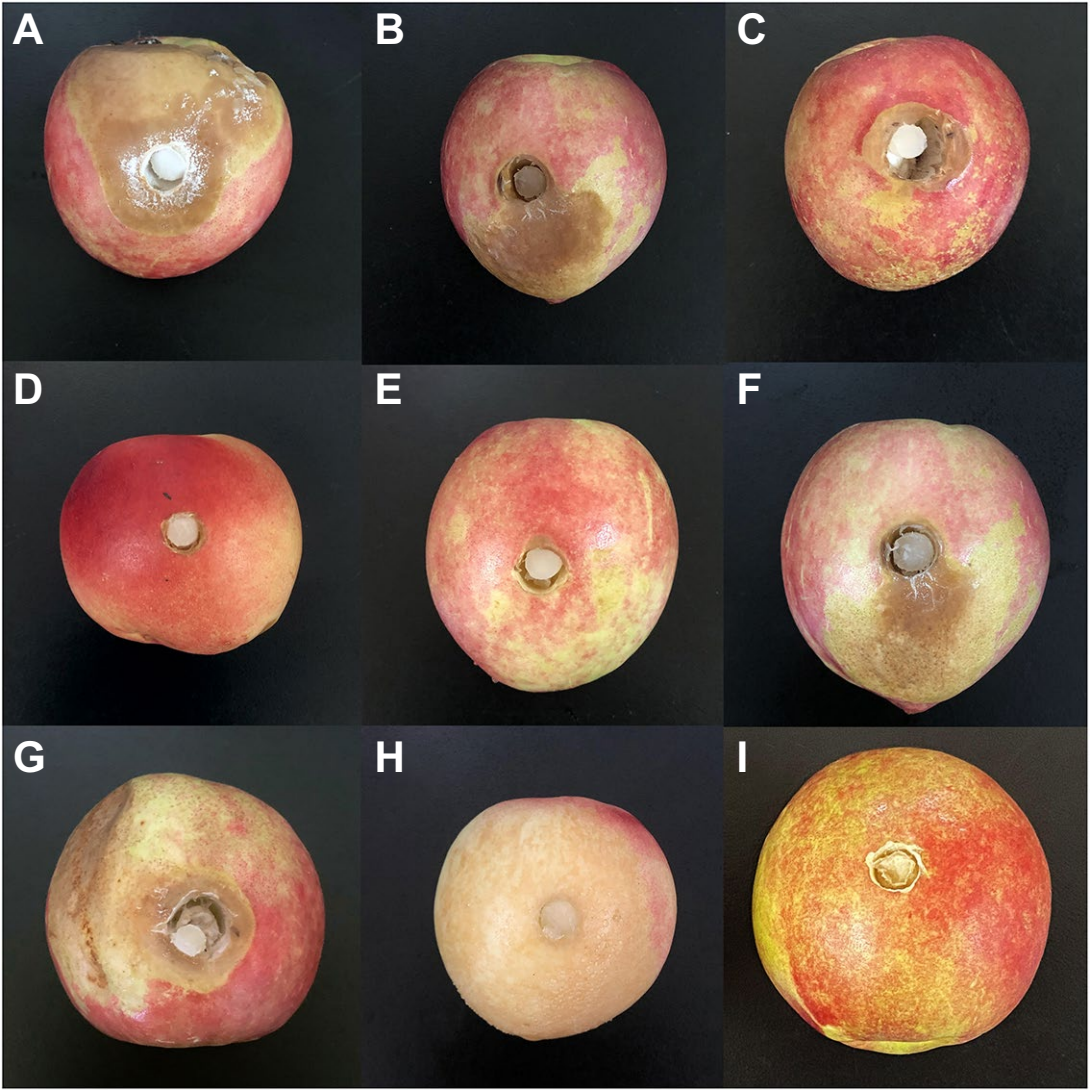
Antifungal activity of WSJK-1and Mby strains and their volatile organic compounds in peaches. **(A)** was control inoculated with *M.fructicola*; **(B)** was treated with WSJK-1 VOCs in 5 days; **(C)** was treated with Mby VOCs in 5 days; **(D)** was treated with compounds of 60 μl L^−1^ 2-Phenylethanol at 5 days; **(E)** was treated with compounds of 60 μl L^−1^ Benzaldehyde at 5 days; **(F)** was treated with compounds of 60 μl L^−1^ (S)- (−) Limonene at 5 days; **(G)** was treated with compounds of 60 μl L^−1^ Limonene at 5 days; **(H)** was treated with compounds of 100 μl L^−1^ Benzaldehyde at 5 days; **(I)** was treated with compounds of 100 μl L^−1^ 2-Phenylethanol at 5 days.

Both WSJK-1and Mby strains had different inhibitory effects on *M. fructicola*. The control effect on *M. fructicola* was 52.31 and 63.11% in peaches fruits (Table 6). After inoculation with *M. fructicola*, all treatments had different degrees of disease in 5 days. In treatment without *C. fimbriata* and VOCs, the peaches were all infected by *M. fructicola* (Figures 3A–C). Benzaldehyde showed the best effect in all compounds with a control effect was 95.89% and peaches were healthy at 60 μl L^−1^ headspace concentration (Figure 3D). However, when the concentration increased to 100 μl L^−1^ the phytotoxicity caused by Benzaldehyde appeared (such as peel browning), and the control effect was 100% (Figure 3H). Similarly, compound 2-Phenylethanol was also effective against *M. fructicola*, the control effect was 86.58% at 60 μl L^−1^ (Figure 3E) and no phytotoxicity occurred even at 100 μl L^−1^ (Figure 3I). 1-Octen-3-ol completely inhibited *M. fructicola* at 20 μl L^−1^, but peel browning occurred in all treatments. Limonene and (S) - (−) Limonene were also able to inhibit *M. fructicola in vivo*, and the control effect was 42.29 and 34.7% (Figures 3F,G).

This result is similar to the biocontrol effect of the previously reported biocontrol strains. Brown rot incidence and severity on peach and plum inoculated with *M. fructicola* were significantly reduced by the biocontrol strains *Debaryomyces hansenii* KI2a and *Wickerhamomyces anomalus* BS91 (92.46, 85.10%). Dimethyl disulfide and Dimethyl trisulfide were the main antifungal compounds of *Pseudomonas fluorescens* ZX strain, they could completely inhibit citrus blue mold at concentrations of 100 and 10 μl L^−1^ *in vivo* (Wang et al., 2021). Isooctanol was the main antibacterial compound produced by *Corrallococcus* sp. EGB. At a concentration of 114.2 μl L^−1^, it could control postharvest blue mold disease caused by *Penicillium digitatum* in citrus and reduce the lesion area by 53% (Ye et al., 2020). After fumigation of mango with 2-Ethyl-5-Methylpyrazine, a VOC of strain *Pseudomonas putida* BP25, for 24 h, the severity of anthracnose on fruits decreased by >76% compared with the control (Archana et al., 2021).

New research demonstrates that microorganisms emit VOCs that trigger robust plant systemic defense responses against pathogenic (Tahir et al., 2017; Sharifi and Ryu, 2018; Sharifi et al., 2022). According to molecular studies, systemic defense responses occur due to an increase in the concentration of metabolites and defense-related enzymes such as phenylalanine ammonia-lyase (PAL) and polyphenol oxidase (PPO; Wu et al., 2019a; Hata et al., 2021; Messa, 2021). The enzyme PAL significantly regulates lignin accumulation and forms defensive structures and product phenols as chemical defenses (Gayoso et al., 2010). PPO and PAL contribute to the resistance against pathogenic microbes. This resulted in a rise in the activities of PAL and PPO in Trichoderma-treated oil palm plants, which led to a reduction in leaf spots (Sunpapao et al., 2018). We compared with the previously reported literature and found that there are compounds in *C. fimbriata* strains that can activate plant induced systemic resistance (ISR), such as Acetoin (Peng et al., 2019), 2-Phenylethanol, and Benzaldehyde (Zhou et al., 2018). Not only a mixture of VOCs but also a single volatile can boost plant defense. Some microorganisms emit Acetoin, 2-Phenylethanol, and Benzaldehyde as VOCs; these volatiles have roles in ISR in plants. For instance, *Bacillus velezensis* GJ11 can produce Acetoin to trigger ISR in plants, pre-treatment with Acetoin is favorable for increasing the peroxidase activity to alleviate the toxic role of ROS (Peng et al., 2019). Therefore, we speculate that the biocontrol mechanism of *C. fimbriata* strain may be: (1) VOCs produced by *C. fimbriata* directly inhibit the growth of pathogenic; (2) VOCs produced by *C. fimbriata* strain can activate the ISR of plants. In future research, we should focus on the biocontrol mechanism of *C. fimbriata* strain.

We observed that under the fumigation of high concentration compounds, the fruit appeared water spots, and the peel softened and browned, indicating the occurrence of physiological damage. It has been suggested that the same molecules at high concentrations may have adverse effects on plant growth, leading to cell necrosis, and resulting in more serious disease symptoms (Dietrich et al., 2010). In our study, compounds 2-Phenylethanol and Benzaldehyde had the best control effect on peach brown rot. At the headspace concentration of 60 μl L^−1^, the compounds could inhibit peach brown rot *in vivo*. 1-Octen-3-ol showed phytotoxicity at the concentration of 20 μl L^−1^, although *M. fructicola* was completely inhibited. The product was completely uncommercial when phytotoxicity occurred, even if there were no fungal diseases in the fruit. This may be due to the high concentration of volatiles, which may directly inhibit the growth of pathogens. It may also be that the effects of volatiles on plant physiology will also affect the development of hyphae or spores. More research is needed.

Our study found that *C. fimbriata* VOCs are safe, with many aromas, and even have a variety of benefits to human health, they are widely used in the production of food additives, food spices, and cosmetics. Including Ethyl acetate, Isobutyl acetate, *n*-Propyl acetate, Butyl acetate, 2-Acetoxyl-3-butanone, 1-Octen-3-ol (S)- (−) Limonene, Limonene, Isoamyl acetate, etc. (Opdyke, 1974; Group, 2009; Api et al., 2017, 2019; Al-Dalali et al., 2019; Lin et al., 2019). 2-Phenylethanol was the main component in rose-based essential oils such as the damask rose (*Rosa damascena* Herrm). The fragrances of 2-Phenylethanol were generally expressed as resembling that of the rose (Oshima and Ito, 2021). Benzaldehyde was an aromatic aldehyde liquid with a typical almond flavor. Benzaldehyde and 2-Phenylethanol have been classified as safe substances by the Food and Drug Administration and are used as flavoring substances to confer almond flavors to foods in the European Union (Andersen, 2006; Wang et al., 2020). Limonene is found in natural fruits like grapefruit, tangerine, orange, mandarine, and lemon. Limonene was frequently used as a dietary supplement and a fragrance ingredient for cosmetics. And Limonene has many benefits for humans, such as hepatoprotective, antidiabetic, anticancer, anti-inflammatory, antioxidant, cardioprotective, gastroprotective, immune-modulatory, anti-fibrotic, anti-genotoxic, and it has antifungal activity in *Trichophyton rubrum* (Chee et al., 2009).

The results showed that the VOCs of *C. fimbriata* inhibited the growth of different fungi. Compared with the chemical fungicides currently used, the fumigation method with VOCs may be more suitable for storing and transporting postharvest fruits and vegetables. VOCs applies not only to different storage stages of various products but also to products that are easily damaged and cannot be treated with liquid fungicides, such as peaches, persimmons, and strawberries. In addition, microbial VOCs easily volatilize and degrade at room temperature and do not easily remain on the food surface. The VOCs produced by *C. fimbriata* are safe. They have been used as food additives and have the special aroma of fruits and flowers. And not contact with fumigated objects has the potential to develop into a new biological fumigant. Thus, it is expected to solve the residue problem of traditional chemical fungicides and their pollution of the environment and water source. So it is a promising application direction for VOCs to prevent postharvest decay as biological fumigation. It is necessary to highlight that even though the findings in this study could provide a new prospect for biological control, further studies, such as VOCs have some limitations in the application, such as being highly sensitive to oxidation, evaporation, and chemical interaction. VOCs, as a fumigant, need a certain amount of time to be effective and VOCs in the field evaporate quickly in the open field (Sharifi and Ryu, 2020). To overcome the limitation of chemicals in nature, encapsulation of VOCs increases their stability and enables their slow release. One practical use of volatiles is through in an encapsulated form (slow-release capsules or microcapsules; Sharifi and Ryu, 2020). Those studies will be done to develop application strategies for commercial production in the future. This study has provided an experimental basis and theoretical support for field experiments and provides necessary information for further using *C. fimbriata* to biological control plant diseases.

## Conclusion

In summary, the *C. fimbriata* strain has diverse VOCs and broad-spectrum antifungal activity, which can inhibit most postharvest pathogens. After fumigation, the mycelium mishappened. Identification of VOCs showed that at least eight compounds had potential antifungal activity. Moreover, most of the compounds have pleasant fragrances and have been recognized by FDA as GRAS compounds, which are widely used in food and spices. *In vivo* tests showed that *C. fimbriata* strain and compounds Benzaldehyde and 2- Phenylethanol had a good control effect on peach brown rot. To the best of our knowledge, this is the first report on the antifungal activity of VOCs produced by *C. fimbriata*. These results show that *C. fimbriata* can be developed as a new biological fumigant and used as a substitute for traditional chemical fungicides for postharvest preservation and storage of fruits and vegetables because of its safety. The antifungal activity mechanism of this strain needs further study.

## Data availability statement

The raw data supporting the conclusions of this article will be made available by the authors, without undue reservation.

## Author contributions

YG and HR: conceptualization, formal analysis, methodology, software, writing-original draft. SH: resources, writing-review & editing. SD, SX and XL: investigation, data curation, formal analysis. QH: designed research, writing-review & editing. All authors contributed to the article and approved the submitted version.

## Funding

We are grateful for the financial support of the National Research Foundation of China (31860522) and National Key Research and Development Program of China (No. 2019YFD1002004).

## Conflict of interest

The authors declare that the research was conducted in the absence of any commercial or financial relationships that could be construed as a potential conflict of interest.

## Publisher’s note

All claims expressed in this article are solely those of the authors and do not necessarily represent those of their affiliated organizations, or those of the publisher, the editors and the reviewers. Any product that may be evaluated in this article, or claim that may be made by its manufacturer, is not guaranteed or endorsed by the publisher.
